# Quality of life: international and domestic students studying medicine in New Zealand

**DOI:** 10.1007/s40037-012-0019-y

**Published:** 2012-06-27

**Authors:** Marcus A. Henning, Christian Krägeloh, Fiona Moir, Iain Doherty, Susan J. Hawken

**Affiliations:** 1 Centre for Medical and Health Sciences Education, University of Auckland, Private Bag 92019, Auckland, 1142 New Zealand; 2 Department of Psychology, AUT University, Private Bag 92006, Auckland, 1142 New Zealand; 3 Goodfellow Unit, School of Population Health, Faculty of Medical and Health Sciences, University of Auckland, Private Bag 92019, Auckland, 1142 New Zealand; 4 ELearning Pedagogical Support Unit, Centre for the Enhancement of Teaching and Learning, University of Hong Kong, Hong Kong, China; 5 Department of Psychological Medicine, Faculty of Medical and Health Sciences, University of Auckland, Private Bag 92019, Auckland, 1142 New Zealand

**Keywords:** Quality of life, World Health Organisation, Medical students

## Abstract

International students form a significant proportion of students studying within universities in Western countries. The quality of life perceptions of international medical students in comparison with domestic medical students has not been well documented. There is some evidence to suggest that international medical students may have different educational and social experiences in relation to their domestic peers. This study investigates the levels of quality of life experienced by international and domestic students studying medicine. A total of 548 medical students completed the abbreviated version of the World Health Organization Quality of Life questionnaire. The focus of the analysis was to evaluate differences between international and domestic students in their early clinical years. The responses were analysed using multivariate analysis of variance methods. International medical students are experiencing lower social and environmental quality of life compared with domestic peers. International medical students in New Zealand have expressed quality of life concerns, which likely have an impact on their academic achievement, feelings of wellness, acculturation, and social adaptation. The findings reinforce the need for creating stronger social networks and accessible accommodation, as well as developing systems to ensure safety, peer mentorship and student support.

## Introduction

The increased globalization of education has enabled the development of an educational industry in New Zealand [1] and overseas [2]. New Zealand is highly valued as an English-speaking destination due to its reasonable cost of living, low student fees, high-quality education, and ease of access to information about courses [1]. Provision of education to international students is also a profitable business enterprise with over 93,000 foreign paying students studying in New Zealand in 2009, contributing to financial gains for the country in the vicinity of 2 billion New Zealand dollars per annum [1]. Contingent on their visa status, students are classified as either domestic or international students (foreign fee-paying students). If the student holds New Zealand citizenship or a residency permit, then the student is classified as domestic, otherwise as international.

Quality of life (QoL) measures are seen as important in providing a comprehensive profile of a person’s health status. QoL has been defined by the World Health Organization working group as: ‘individuals’ perceptions of their position in life in the context of the culture and value systems in which they live and in relation to their goals, expectations, standards and concerns’ (p. 551) [3]. This definition tends to focus on aspects of health and well-being and complements more objective measures related to functional health status [4]. A commonly used instrument measuring QoL is the abbreviated version of the World Health Organization QoL questionnaire (WHOQOL-BREF), which employs the domains of physical health, psychological health, social relationships and environment [3, 4]. There are numerous other measures of health-related QoL cited in the literature, each with their own strengths and limitations according to the criteria of feasibility, validity, responsiveness and interpretability [5]. The WHOQOL-BREF has been systematically appraised across numerous cultural groupings [6, 7] and was considered to be suitable for the medical student group [8].

QoL, well-being and mental health issues have been studied in reference to student populations [9] including medical students [10–12]. Leahy et al. [9] measured psychological distress in students in Australia and found that students from all tertiary disciplines expressed levels of distress which were higher than age-matched peers within the general population. Explanations for this difference range from financial commitments, intensive study regimes and family obligations [9]. However, not all comparative studies have concluded that tertiary students have higher levels of mental ill-health than their non-student peers. One large US study compared the 12-month prevalence of psychiatric disorders (by diagnostic interviews) in students and non-student peers and found that there was no difference [13].

It is clear that the mental health and well-being of medical students has become a prominent issue over the last few years [11, 12, 14–16]. Some of the major issues cited are related to psychopathology [11, 14], the pressures of the medical learning environment, and external factors affecting student well-being such as debt and transitioning into the clinical environs [11, 12, 15, 16]. In New Zealand, Henning et al. [10] found that medical students early in their clinical training experienced sleep problems, and felt anxious and uncertain in the clinical setting. Moreover, a recent systematic review of student mental health reported that studying at medical school was often associated with high levels of stress [17]. Interestingly, none of these studies have focussed on specific concerns related to international students.

To address concerns related to wellness amongst students, a *guild* was formed in the United Kingdom to further develop the promotion of mental well-being in higher education [18]. The formation of this guild was driven by alterations being made to disability legislation and communities being at risk of social exclusion. Similarly in Australia, recent guidelines [19] have been developed for tertiary education institutions to ‘facilitate improved educational outcomes for students with a mental illness’ (p. 1), as it is clear that the ability to deal with emotional stress has an impact on academic performance and the successful completion of a qualification [17]. The report by the Royal College of Psychiatrists in London: ‘*Mental Health of Students in Higher* *Education*’ summarized the challenges specific to international students. These challenges included adjusting to a new cultural and academic environment, financial constraints impeding regular visits to family overseas, lack of English language skills affecting academic achievement, and the pressure of expectations from self and others [18]. It has been shown that international students, when making decisions to study abroad, may consider safety and security, the international reputation of the university, cost of living, visa and entry requirements, and QoL issues [20].

Using the WHOQOL-BREF, Chai et al. [21] found that international students studying courses other than medicine at a New Zealand university rated their experiences of physical and environmental QoL lower than domestic students but found no differences with respect to psychological and social relationships. Using a similar sample, Hsu et al. [22] found that international students rated physical and environmental QoL lower than domestic students. In addition, Asian international students studying medicine in New Zealand were found to be more anxious about tests, whilst also scoring lower on environmental QoL than their domestic Asian peers [23]. These results are consistent with findings presented in studies conducted in other countries [24, 25]. For example, Sam [25] incorporated a life satisfaction measure and surveyed international students studying in a Norwegian university. In Sam’s study, African students rated life satisfaction lower than expected in relation to normative data but other international students were within normative expectations. Lee et al. [26] suggested that one reason why international students may have greater difficulties with quality of life when studying abroad is that they experience ‘acculturative stress’ or stress resulting from the strain of educational and social adaptation.

There appears to be a wealth of data about international students in general, but very little related to the medical student context. Consequently, the aim of the present paper is to explore the levels of QoL experienced by students studying medicine in New Zealand, and to compare data from international and domestic student groups. It was expected that international students are likely to encounter more challenges in terms of their QoL.

## Method

### Participants

This study examined the responses from 548 medical students early in their clinical training (Years 4 and 5) from two different time periods (2009 and 2011) studying at the University of Auckland, where one of New Zealand’s two medical schools is located. More detailed demographic information and response rates are shown in Table 1.

**Table 1:** Demographic details of participants (*n* = 549)

	Year of study	
	2009	2011
Response rate (%)	80	78
Age		
Years (*SD*)	22.74 (2.75)	22.86 (2.65)
Cohort	273	275
Gender		
Male	122	118
Female	150	156
Study year		
Year 4	165	127
Year 5	108	148
Ethnicity		
Asian	97	89
European	99	113
Maori	14	7
Pacific island	14	10
Other	47	54
Enrolment status		
Domestic	225	243
International	49	32

*Note* Some respondents did not provide all details resulting in missing values for some variables

### Procedure

Data collection for this study was conducted during academic semesters in 2009 and 2011. Students were asked to fill in a demographic survey and the Australian version of the WHOQOL-BREF [28]. Ethics approval was gained from the University of Auckland Human Participants Committee. After permission from senior faculty was obtained, the researchers introduced the purpose and nature of the study to fourth and fifth year medical students immediately after or prior to a scheduled lecture time. Students were invited to fill in the paper-based questionnaire, which was then collected by the researcher and assistants. The questionnaires took approximately 10–15 min to complete.

### Measures

The WHOQOL-BREF has 26 items; this includes two global items about health-related QoL and 24 items relating to four domains (physical, psychological, social, and environmental QoL). The respondents rate the items using a five-point Likert scale; except for three negatively worded items, a low rating towards 1 suggests a negative evaluation, and a high rating towards 5 indicates a more positive perception of QoL.

Enrolment status (international; domestic) was identified as the independent variable of interest. However, other variables were considered in terms of their potential influence such as time of investigation (2009; 2011), gender, and year of enrolment (year 4; year 5). Age was included as a covariate. Ethnicity was only examined in the initial analyses as nearly all the international students were classified as Asian or other (*n* = 79, 98 % of international students). Unpublished departmental statistics show that the majority of international students enrolled were classified as Malaysian (78 % in 2009 and in 75 % in 2011).

### Data analysis

For this study, the multivariate analysis of covariance (MANCOVA) approach was predominantly utilized with the statistical package PASW version 18 [29]. The main analyses were conducted at the domain level. The physical domain scores were determined by averaging the scores on seven items inquiring about physical quality of life, the psychological domain using the average of six relevant items, the social domain three items, and the environmental domain eight items. Other analyses were conducted at the facet level, or in other words, using individual items. Cohen’s *d* effect sizes and 95 % confidence intervals [30, 31] were also calculated for the domain and facet score differences. Thalheimer and Cook [31] stated that effect sizes of around 0.2 are deemed ‘small’, 0.5 ‘medium’, and 0.8 ‘large’. Remaining analyses included Cronbach’s alpha, Bonferroni’s correction and correlational comparisons.

## Results

### Preliminary analyses

All WHOQOL-BREF domains exhibited high levels of internal consistency (α_physical_ = 0.76, α_psychological_ = 0.82, α_social relationships_ = 0.72, α_environment_ = 0.76). A precursor MANCOVA showed no differences between year 4 and 5 students’ QoL domain scores; hence, this variable was not included in the subsequent multivariate analyses.

### Multivariate analyses

The multivariate statistical analyses revealed significant main effects for ‘*enrolment status*’ (International; Domestic) [*F*(4, 521) = 5.17, Wilks’ lambda = 0.96, *p* < 0.01]. Further main effects were noted for ‘*cohort*’ (2009; 2011) [*F*(4, 521) = 4.31, Wilks’ lambda = 0.97, *p* < 0.01], and ‘*gender*’ (Male; Female) [*F*(4, 521) = 2.83, Wilks’ lambda = 0.98, *p* < 0.05]. The covariate *age* also yielded a significant result [*F*(4, 521) = 2.85, Wilks’ lambda = 0.98, *p* < 0.05]. No significant interaction effects were noted.

### Between-group analyses: WHOQOL-BREF domains

The between-group analyses (Table 2) yielded five significant main effects in terms of the WHOQOL-BREF domains.

**Table 2:** Tests of between-subjects effects for cohort, enrolment status, and gender over the four WHOQOL-BREF domain measures with age and year of study as covariates

Source	Dependent variable	*MS*	*F*	*df*_1_	*df*_2_
Covariate (age)	Physical	1.46	5.02*	1	524
	Psychological	0.11	0.29	1	524
	Social	0.50	0.87	1	524
	Environment	2.42	7.82*	1	524
Enrolment status (ES)	Physical	1.12	3.83	1	524
	Psychological	0.95	2.46	1	524
	Social	7.33	12.78**	1	524
	Environment	3.32	10.71**	1	524
Cohort (CO)	Physical	3.96	13.59**	1	524
	Psychological	0.44	1.13	1	524
	Social	0.22	0.39	1	524
	Environment	0.20	0.66	1	524
Gender (GE)	Physical	1.00	3.45	1	524
	Psychological	2.75	7.13*	1	524
	Social	0.08	0.14	1	524
	Environment	1.92	6.19*	1	524
ES × CO	Physical	1.86	6.37*	1	524
	Psychological	0.54	1.41	1	524
	Social	0.21	0.37	1	524
	Environment	0.01	0.02	1	524
ES × GE	Physical	0.10	0.36	1	524
	Psychological	0.17	0.45	1	524
	Social	0.57	0.99	1	524
	Environment	1.17	3.79	1	524
CO × GE	Physical	1.31	4.50*	1	524
	Psychological	0.12	0.32	1	524
	Social	0.27	0.46	1	524
	Environment	0.46	1.48	1	524
ES × CO × GE	Physical	0.53	1.80	1	524
	Psychological	0.24	0.61	1	524
	Social	0.00	0.00	1	524
	Environment	0.86	2.77	1	524

* *p* < 0.05, ** *p* < 0. 01

1. Enrolment status—two significant main results with respect to the social relationships domain [*F*(1, 524) = 12.78, *p* < 0.01] and the environment domain [*F*(1, 524) = 10.71, *p* < 0.01] (see Table 3 for mean comparisons).

**Table 3:** Means (and standard deviations) with Cohen’s *d* values (and confidence intervals) for the WHOQOL-BREF domains and facets with respect to cohort, enrolment status and gender

Scale points/domains and facets	Enrolment status		Effect size measures		
	Domestic (*n* = 461)	International (*n* = 79)	Cohen’s *d*	95 % CI for Cohen’s *d*	
				Lower	Upper
1. Physical health	4.02 (0.55)	3.85 (0.56)*	0.31	0.07	0.55
Pain and discomfort	4.54(0.73)	3.89 (1.07)**	0.82	0.58	1.07
Dependence on medication	4.55 (0.74)	4.52 (0.68)	0.04	−0.20	0.28
Energy and fatigue	3.55 (0.86)	3.49 (0.73)	0.07	−0.17	0.31
Mobility	4.43 (0.80)	4.03 (0.95)**	0.49	0.24	0.73
Sleep and rest	3.39 (1.07)	3.52 (1.06)	−0.12	−0.36	0.12
Activities of daily living	4.00 (0.83)	3.83 (0.81)	0.21	−0.03	0.44
Work capacity	3.68 (0.92)	3.64 (0.88)	0.04	−0.19	0.28
2. Psychological	3.64 (0.64)	3.50 (0.54)	0.22	−0.02	0.46
Positive feelings	3.95 (0.76)	3.65 (0.70)*	0.40	0.16	0.64
Meaningfulness of life	3.88 (0.91)	3.73 (0.83)	0.17	−0.07	0.41
Thinking, learning and concentration	3.22 (0.79)	3.19 (0.72)	0.04	−0.20	0.28
Body image	3.64 (0.93)	3.52 (0.97)	0.13	−0.11	0.37
Self-esteem	3.73 (0.88)	3.60 (0.81)	0.15	−0.09	0.39
Negative feelings	3.39 (0.93)	3.30 (0.91)	0.10	−0.14	0.34
3. Social relationships	3.82 (0.76)	3.47 (0.71)**	0.46	0.22	0.70
Personal relations	3.83 (0.94)	3.47 (0.97)*	0.38	0.14	0.62
Sex	3.63 (1.06)	3.16 (0.98)**	0.45	0.21	0.69
Social support	3.98 (0.81)	3.76 (0.85)*	0.27	0.03	0.51
4. Environment	3.81 (0.58)	3.57 (0.47)**	0.42	0.18	0.66
Physical safety and security	4.25 (0.75)	4.00 (0.62)**	0.34	0.10	0.58
Physical environment	3.96 (0.82)	3.75 (0.76)*	0.26	0.02	0.50
Financial resources	3.28 (1.17)	3.57 (0.98)*	−0.25	−0.49	−0.01
Information and skills	3.84 (0.79)	3.61 (0.69)*	0.30	0.06	0.54
Recreation and leisure	3.36 (0.91)	3.15 (0.94)*	0.23	−0.01	0.47
Home environment	4.06 (0.91)	3.76 (0.84)*	0.33	0.09	0.57
Access to health services	3.95 (0.95)	3.37 (0.88)**	0.62	0.37	0.86
Transport	3.81 (1.06)	3.35 (1.06)**	0.43	0.19	0.67

* *p* < 0.05, ** *p* < 0.01
2. Cohort—one significant main result with respect to the physical domain [*F*(1, 524) = 13.59, *p* < 0.01].
3. Gender—two significant main results with respect to the psychological domain [*F*(1, 524) = 7.13, *p* < 0.05] and the environment domain [*F*(1, 523) = 6.19, *p* < 0.05], with male students (*M*_psychological_ = 3.64, *SD*_psychological_ = 0.55; *M*_environment_ = 3.83, *SD*_environment_ = 0.59) scoring higher than female peers (*M*_psychological_ = 3.52, *SD*_psychological_ = 0.52; *M*_environment_ = 3.74, *SD*_environment_ = 0.55) on both domains.

A significant interaction effect was noted for cohort by enrolment status [*F*(1, 524) = 6.37, *p* < 0.05] for physical QoL. The means scores suggest a marked difference for physical QoL in reference to the 2009 cohort (*M*_international_ = 3.69, *SD*_international_ = 0.54; *M*_domestic_ = 3.97, *SD*_domestic_ = 0.57) when compared with the 2011 cohort (*M*_international_ = 4.08, *SD*_international_ = 0.52; *M*_domestic_ = 4.06, *SD*_domestic_ = 0.53), which did not show a noticeable difference.

A further incidental interaction was noted for cohort by gender [*F*(1, 524) = 4.50, *p* < 0.05]. No other significant results were found.

### Facet scores

To gain more specificity to the analysis, facet differences (see Table 3 for details) between the international and domestic students were investigated within each of the WHOQOL-BREF domains. Potential confounding variables (cohort, gender and age) were also entered into the analytical model. The means and standard deviations were compared employing a MANCOVA approach followed by a series of univariate tests on facets within each domain. Cohen *d* measures were also generated to estimate effect size differences for each comparison.

From the possible 24 facet differences (Table 3), 14 facets yielded significant differences. Ten of the possible 11 facet differences for social relationships and environment were identified:

1. In the social relationships QoL domain, domestic students rated ‘personal relations’ and ‘sex’ more positively than their international peers.
2. In the environment QoL domain, domestic students rated ‘physical safety and security’, ‘physical environment’, ‘information and skills’, ‘recreation and leisure’, ‘home environment’, ‘access to health services’, and ‘transport’ higher than their international peers. However, domestic students rated ‘financial resources’ lower than their international peers. Other differences were also noted in relation to ‘pain and discomfort’, ‘mobility’, and ‘positive feelings’. In each of these differences domestic students out-rated their international peers, suggesting higher levels of QoL according to these facets. The univariate results were in agreement with the effect size measures.

### Ethnicity and enrolment status

It was noted that 98 % of international students were self-classified as either Asian (*n* = 62) or ‘Other’ (*n* = 17). No differences or interactions were found between the Asian and ‘Other’ cohort [*F*(4, 255) = 1.40, Wilks’ lambda = 0.98, *p* > 0.05] in terms of the WHOQOL-BREF measures; controlling of gender, cohort, age and enrolment status.

When investigating differences between international and domestic students within the Asian student cohort, one significant difference was obtained for environmental QoL, with domestic Asian students scoring higher (*M* = 3.81, *SD* = 0.57) than their international Asian peers (*M* = 3.58, *SD* = 0.49); indicating higher levels of QoL for the domestic group.

## Discussion

The present investigation aimed to explore QoL issues encountered by international and domestic medical students. Several important differences were noted in this study. First, international students expressed different QoL perceptions compared with their domestic student counterparts. Second several significant results were noted in relation to the student cohorts, gender, and ethnicity.

### International versus domestic students

It has been established that international students may be more at risk in reference to their QoL than their domestic peers [21–23, 26]. However, the characteristics of these two groups and associations with QoL have not been investigated in medical students. The findings from this study showed that domestic students rated their social and environmental QoL higher than international students. A further finding was the general lack of interaction found between enrolment status, gender and cohort, suggesting that international students are encountering the same problems in 2011 that they faced in 2009, and in terms of enrolment status male and female students have similar issues. Nonetheless, there was one interaction effect noted for physical QoL between cohort and enrolment status which is discussed below.

Further investigations at the facet level revealed differences in terms of social relationships, whereby domestic students rated both their level of satisfaction with their personal relationships, social support and sex life higher than international students. More strikingly, differences were noted according to environmental QoL perceptions, in which domestic students felt more secure and safe, lived in more healthy environments, were more able to access information relevant to their day-to-day living, had more opportunity for leisure activities, and were more satisfied with transport, home environment and access to health services when compared with the perceptions of international students. There is some evidence to suggest that international students within New Zealand do experience more alienation due to problems associated with transportation and the wide geographical area of Auckland (the largest city in New Zealand) [31]. Furthermore, Sam [25] found no difference in measures of satisfaction with life when comparing domestic and international students in Norway except for students of African origin. There are, therefore, likely to be unique contextual factors when explaining differences of this kind. One way to progress this supposition would be to consider some of the unique characteristics of each country in association with some global factors such as the ability to communicate fluently in the host language [32].

A way forward in addressing the needs of international students is to consider their reasons for choosing overseas destinations for education. Cubillo et al. [20] developed a theoretical model suggesting that students choose to study abroad because of personal reasons, the image of the country and city, reputation of the institution and programme of study. To adequately plan for study in distant educational facilities, students often go through several decision processes that include choosing the course, investigating entry pathways into different courses, considering alternatives and then deciding on entry characteristics [33]. The findings of the present study suggest that there are some uniform problems with international students and that the source may be environmental rather than intrinsic and these environmental factors may heighten levels of acculturation stress [26]. Finding solutions for environmental concerns may be easier to attend to than other problematic conditions. For example, providing greater regulation of ‘homestay’ situations (living with host families) and developing more socially supportive environments would both go some way to improving the environmental conditions of international students [34]. Providing comprehensive information about not only the university course but also the surrounding areas may also be useful [33]. In addition, providing orientation courses and peer mentors may be another method to alleviate acculturation stress [35]. Although not investigated, language [26, 36] is unlikely to be a major problem for the international students within this cohort given the high level of language competency required to study medicine.

One environmental QoL area that was found to be significant in favour of international students is finances. International students appeared to be more satisfied when asked about access to enough money to meet their needs. This difference is likely related to domestic students reliance on loans and thus acquiring a large student debt by the end of their study in addition to the costs associated with day-to-day living [37, 38] and the likely impact on well-being [39]. There is some evidence to suggest that whilst international medical students incur larger enrolment fees, they are more likely to come from affluent backgrounds [40]. They may also have international scholarships or have stringent entry criteria in relation to access to funding [41]. Unpublished departmental statistics for this cohort of students showed that the majority of Asian students studying at this University came from Malaysia and it is likely that about 50 % of these were being financed via a scholarship. However, some studies have indicated that international students do in some cases incur finance-related problems [42, 43], and that crisis situations for international students may have some association with financial difficulties [36]. In addition, there may be an inherent response bias in terms of revealing information about financial hardship [23, 34].

### Cohort (2009 vs. 2011)

The results of the present study also indicate a difference between the 2009 and 2011 cohorts in terms of their perceptions of physical QoL with a significant interaction noted for enrolment status. This finding revealed that there was a substantial difference in self-related physical QoL between international and domestic students in the 2009 cohort but not in the 2011 cohort. The issue of wellness has been increasingly and proactively addressed within this University in the Faculty of Medical and Health Sciences. One such initiative was developed and made available to students in December 2008. This website [44] (which is publicly available) was developed to promote strategies and awareness with regards issues related to mental resilience, managing stress, anxiety and depression, healthy relationships, and finding meaning in life. It may be that initiatives such as this website [44] have begun to have an impact on the culture of teaching and learning within this University resulting in a more university-wide emphasis in the last few years in relation to addressing quality of life issues.

### Gender and ethnicity

The lack of interactions found in the results of this study indicated that the subgroups of international students (gender and ethnicity) do not have a significant impact on the direction of international students’ QoL perceptions. This is somewhat contrary to some literature suggesting that female international students are experiencing a greater sense of vulnerability than other student groups including male international students [31].

Of the international students in the present sample, 77 % were classified as Asian. When the Asian international group was compared with ‘other’ international students no differences were found. Additionally, when the domestic Asian group was compared with their international peers, the domestic students rated significantly higher in relation to environmental QoL indicating that enrolment status has more impact on QoL perceptions than ethnicity. This may, in part, be related to Auckland’s transportation and accommodation problems [31] when compared with European cities [25] which are likely to have more accessible transportation and be more similar to Asian cities in this respect. In addition, several studies have found similar differences between Asian and European students within the Auckland setting [21, 23].

### Limitations

Several limitations of the study need to be acknowledged. Firstly, there is always the possibility of type 1 error (claiming a significant result when one does not in reality exist) when considering differences between several means being evaluated in tandem. However, given the extent and direction of the differences found in this study we believe that the risk of type 1 error is minimal. Secondly, the two groups were not randomized; however, given the high response rates we believe problems associated with quasi-experimental designs to be negligible. Lastly, there is a possibility that response bias may exist given the cultural differences between the international and domestic groups and this may need to be explored in further research.

## Conclusion

The findings of this study suggest that the international medical students differ from the domestic students in terms of their experience of quality life. There is some evidence that quality of life does have an impact on academic achievement [45, 46], which suggests that international students are likely to be experiencing greater study stress than domestic students. It is also likely that international students within the present study population are experiencing more psychological problems such as depression and anxiety as documented elsewhere [11, 12, 26, 36]. Mechanisms for minimizing acculturation stress need to be considered at both university and community levels, such as developing more amenable and accessible student accommodation schemes.

Additionally, the assumption in New Zealand, as in other Western countries, is that New Zealanders are good hosts and take care of their overseas visitors and students [47]. There is some evidence to support this claim but there are also instances of racial discrimination and poor communication within ‘homestay’ situations [34]. These inconsistencies highlight the need to put in additional measures to address quality of life imbalance between domestic and international students and to address any instances of international students experiencing implicit and/or explicit abuse. Such solutions may include creating stronger social networks, more accessible accommodation, systems to ensure safety and security, and developing peer mentor mechanisms.
